# Evaluation of conventional, protaper hand and protaper rotary instrumentation system for apical extrusion of debris, irrigants and bacteria- An *in vitro* randomized trial

**DOI:** 10.4317/jced.53340

**Published:** 2017-02-01

**Authors:** Pinky Kalra, Arathi Rao, Ethel Suman, Ramya Shenoy, Baranya-Shrikrishna Suprabha

**Affiliations:** 1 Ex-Post Graduate Student, Paedodontics & Preventive Dentistry, Manipal College of Dental Sciences, Mangalore, Manipal University, Karnataka State, India; 2 Professor & Head, Paedodontics & Preventive Dentistry, Manipal College of Dental Sciences, Mangalore, Manipal University, Karnataka State, India; 3 Associate Professor, Microbiology, Kasturba Medical College, Mangalore, Manipal University, Karnataka State, India; 4 Associate Professor, Public Health Dentistry, Manipal College of Dental Sciences, Mangalore, Manipal University, Karnataka State, India; 5 Professor, Paedodontics & Preventive Dentistry, Manipal College of Dental Sciences, Mangalore, Manipal University, Karnataka State, India

## Abstract

**Background:**

Endodontic instrumentation carries the risk of over extrusion of debris and bacteria. The technique used and the type of instrumentation influences this risk.

**Aim:**

The purpose of this study was to evaluate and compare the K-file, ProTaper hand and ProTaper rotary instrumentation systems for the amount of apically extruded debris, irrigant solution and intracanal bacteria.

**Design:**

Experimental single blinded randomized type of in vitro study with sample of 30 single rooted teeth. Endodontic access cavities were prepared and the root canals were filled with the suspension of *E. faecalis*. Myers and Montogomery Model was used to collect apically extruded debris and irrigant. Canals were prepared using K files, Hand protapers and Protaper rotary files.

**Statistical analysis:**

Non Parametric test like Kruskal-Wallis and Mann-Whitney U test were applied to determine the significant differences among the group.

**Results:**

Tests revealed statistically significant difference between the amount of debris and number of bacteria extruded by the ProTaper hand and the K-files. No statistically significant difference was observed between the amounts of irrigant extruded by the ProTaper hand and the K-file system. Statistically significant differences were observed between the amounts of bacteria and irrigant extruded by the ProTaper rotary and the Protaper hand. No statistically significant difference was observed between the amounts of debris extruded by the ProTaper hand and the K-file system.

**Conclusions:**

Amount of apical extrusion of irrigant solution, bacteria and debris are significantly greater with K File instruments and least with Protaper rotary instruments.

** Key words:**Protaper, rotary, periapical extrusion.

## Introduction

Root canal treatment aims at thorough debridement and shaping of the root canal to eliminate viable bacteria and toxins from the root canal and achieve complete 3D obturation so as to sustain adequate periradicular health (1,2). The inter-appointment flare-up is a complication resulting in pain, swelling or both, which is usually seen within a few hours or days after initiation of root canal treatment. Cause for such a flare up is said to be due to apical extrusion of debris and bacteria during instrumentation and studies have shown that almost all instrumentation techniques produce apical extrusion of debris to some extent (3).

An instrumentation technique that minimizes apical extrusion of debris would be advantageous. Many factors such as improper technique of irrigation, excessive instrumentation, the type of file used may affect the amount of apical extrusion (4-6). Various instrumentation techniques have been advocated to minimize the extrusion of debris apically. Conventionally hand filing with K-files were employed, which extruded considerable amount of debris. With advances in preparation and instrumentation techniques (7), it is therefore important to identify the techniques which reduces the extrusion of the debris in an apical direction. The purpose of the study was thus to evaluate apical extrusion during endodontic preparation using three instruments techniques.

The objective was to quantitatively evaluate and compare the amount of apical extrusion of dentinal debris, volume of irrigant solution and intracanal bacteria following canal preparation using K file, Protaper hand and Protaper rotary technique.

The null hypothesis tested was that Protaper rotary instruments caused maximum extrusion of debris and irrigant solution.

## Material and Methods

This was an *in vitro* single blinded study with lottery method of randomization and was approved by Institutional Ethics Committee.

•Inclusion Criteria

Non carious intact premolars with single apical foramen.

•Exclusion Criteria

Premolars with immature root, tooth with fractured root, multiple canals, calcified canals, root caries or any other gross developmental abnormalities and this was confirmed using Operating Loupes (Magni Vision, Confident, India)

-Sample size and selection

The sample size was estimated to be total 30 with 10 in each group considering a study power of 95%

The teeth were allocated into three groups by lottery method of randomization. The groups were as follows: Group 1: K file (Densply Co, India) n=10; Group 2: Protaper Hand (Densply Co, India) n=10; Group 3: Protaper Rotary (Densply Co, India) n=10.

-Test apparatus

A model system as described by Myers and Montogomery 1991 *et al.* (8) was to evaluate bacterial extrusion. The amber colored glass vials with rubber stoppers were used. Holes were created in the rubber stoppers of vials with a hot instrument. The tooth was inserted under pressure into rubber cap till the level of cement-enamel junction.

**Figure 1:**
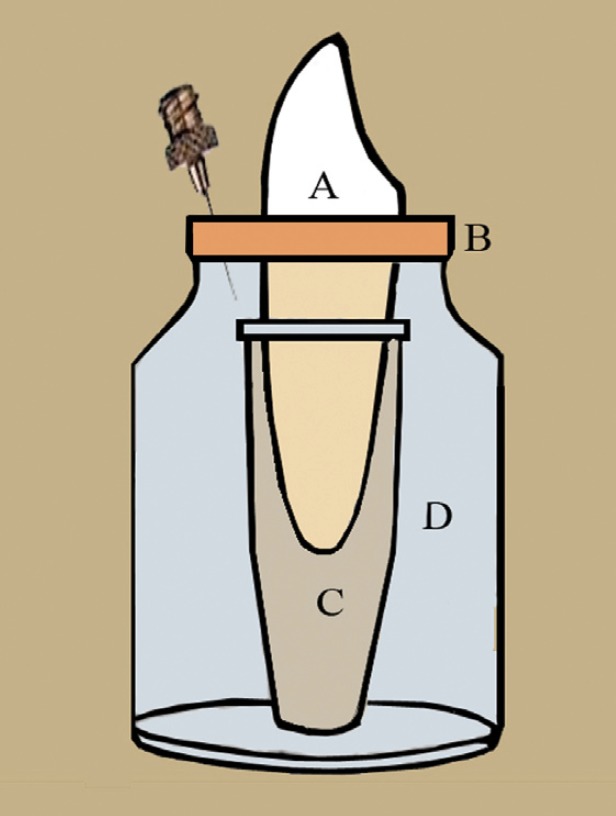
Apparatus to collect the debris. A –Tooth; B – Rubber cap; C – Collecting vial; D – Glass vial; E –22 Gauge needle.

The tooth with the rubber stopper was then fitted into the mouth of the vial. The collecting vials were placed inside the glass vial and the apical part of the root was suspended within the collecting vial to capture and hold the extruded material through the apical foramen. The vials were vented with a 24-gauge needle on the top to equalize the air pressure inside and outside the vial. The apparatus was sterilized in autoclave at 120◦C at 15lbs for 15 minutes. The collecting vials were pre weighed by electronic balance, (Fig. 1):

-Tooth preparation

Three coats of nail varnish was applied to the external surface of all the roots to prevent microleakage from external canals.

Endodontic access cavities were prepared with Endo Access Bur using a high-speed hand piece. Pulp remnants were extirpated with a fine barbed broach. A sterile 10 K file was pushed 1mm beyond the apical foramen to create a hole in the nail varnish coat that covered the apical foramen. In this way a standard size of foramen and apical patency was achieved. The access cavity was used to create a reservoir for loading a suspension of *E. faecalis* (ATCC 29212).

1ml of pure culture of *E. faecalis*, grown in brain heart infusion broth (24h culture) was used. Turbidity was adjusted to 0.5 MacFarland standard to ensure that number of bacteria was 1.5 x 108 CFU/ml. A 10 K file was used to carry down the bacteria to the entire length of canal. The root canals were then dried in an incubator at 37ºC for 24h (9).

-Root canal preparation

Root canal preparation was done by a single operator, using aseptic techniques under a class I laminar airflow to prevent bacterial contamination.

The instrumentation sequences used were as follows:

1. Group 1 (K- file): Gates glidden was used to prepare the access and to enlarge the coronal orifice and prepare the coronal and middle third of the canal. The apical part was prepared by using K files from no. 10 to no. 40 file in sequential order.

2. Group 2 (Protaper hand): Specimens were prepared with Protaper Hand files in a crown down manner according to manufacturer’s instructions using a gentle in-and-out motions. The shaping file SX was used first and moved apically to 2mm short of the working length, followed by S1and S2 for shaping the coronal two-third of the canal. The apical one-third of the canal was finished using F1, F2, and F3 sequentially to the working length.

3. Group 3 (Protaper rotary): The specimens were prepared with Protaper rotary files in a crown down manner according to manufacturer’s instructions using a gentle in-and-out motions. The shaping file SX was used first and moved apically to 2mm short of the working length, followed by S1 and S2 for shaping the coronal two-third of the canal. The apical one-third of the canal was finished using F1, F2, and F3 sequentially to the working length. Once the instrument negotiated the end of the canal and rotated freely, it was removed.

The working length was estimated by subtracting 1mm from the canal length. Root canals irrigants were used between each file with 3ml of distilled water using 22 gauge needle passively placed down the canal extending upto 3 mm from the apical foramen without binding.

The solution was collected at the end of the preparation and thoroughly mixed in a Vortex mixer (CM 101 Cyclomixer, Remi, India) for 5 minutes. The debris were then allowed to sediment for 10 minutes. The volume of extruded irrigant solution was measured using micropipette of 200 µl and 20 µl size. 0.01ml of apically extruded irrigant was taken from collecting vial to count the bacteria. The suspension was plated on brain heart infusion agar at 37ºC for 24h and counting of bacteria was done by Surface viable count by spreading method in triplicate. Viable count was calculated from the average colony count/plate. Debris adhering to outer surface of root apex was collected by washing the apex with 1ml of distilled water in collecting vial. Collecting vials were kept in an incubator at 37ºC till the irrigant had evaporated. The debris was weighed on electronic balance of 10-5 precision. Three consecutive readings were noted for each sample and average value was recorded.

Apical extruded debris = Post operative weight - Pre operative weight of collecting vial.

The bacterial testing and quantitative estimation of debris and irrigant was done by a second investigator who was blinded to the groups.

-Statistical analysis 

Data analysis was carried out by SPSS16.0 Software. Descriptive statistics for extruded debris, irrigant solution and bacteria were calculated by using mean and standard deviation. The Non Parametric test like Kruskal-Wallis and Mann-Whitney U test were applied to determine the significant differences among the group. *p*< 0.5 was taken as statistically significant.

## Results

The mean extrusion values and standard deviation (SD) for each group are presented in  Table 1. Extrusion of irrigant, debris and bacteria were assessed using the Independent Kruskal-Wallis Test.

**Table 1:**
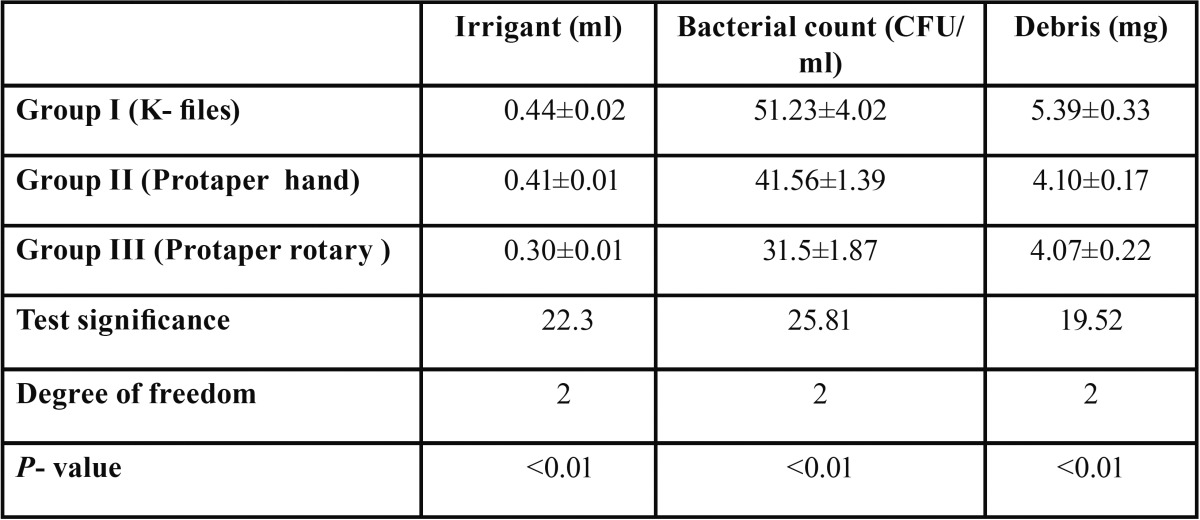
Descriptive statistics and association between extrusion of irrigant, debris and bacteria (Independent Kruskal-Wallis Test).

Irrigants, debris and bacterial extrusion was least with rotary protaper followed by hand protaper and highest with K files. It was statistically significant (*p*-value <0.01).

Mann-Whitney tests were applied for intergroup comparison. Statistically significant difference were observed between the amounts of debris and number of bacteria extruded by the Protaper hand and the K-files (*p*< 0.01).

No statistically significant difference was observed between the amounts of irrigant extruded by the Protaper hand and the K-file system. Statistically significant difference were observed between the amounts of bacteria and debris and irrigant extruded by the Protaper rotary and the K-files (*p*< 0.00). Statistically significant differences were observed between the amounts of bacteria and irrigant extruded by the ProTaper rotary and the Protaper hand with (*p*< 0.00). No statistically significant difference was observed between the amounts of debris extruded by the Protaper hand and the K-file system ( Table 2).

**Table 2:**
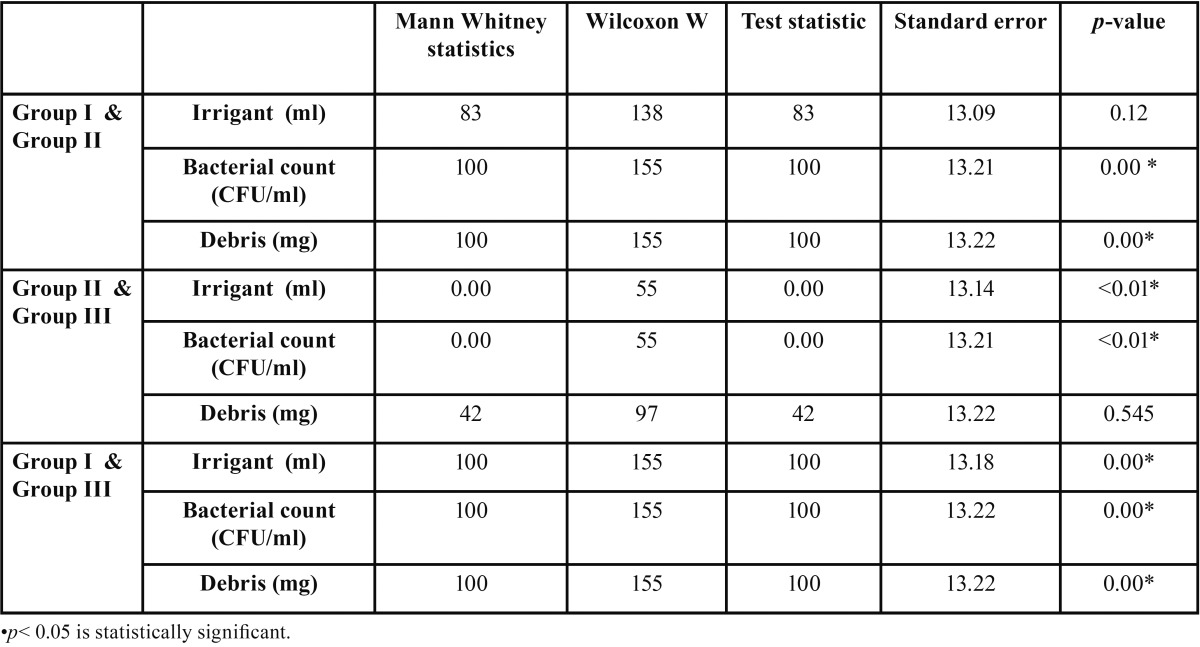
Comparison of apical extrusion of irrigant, bacterial count, debris between group I (K-files) and group II (Protaper hand) and group III (Protaper rotary).

## Discussion

Premolars with single apical foramen were selected for the study as the presence of more than one canal may affect the final amount of apical extrusion (10).

The model described by Myers & Montgomery (8) simulates a clinical working environment where the operator is dependent on working length determination without seeing the root canal space and also prevents bias by the practitioner (11). A standard tooth model increases the probability that the amount of apically extruded bacteria was a result of instrumentation technique and not due to the tooth morphology (12,13).

The working length was maintained at 1 mm short of the apical foramen. Martin and Cunningham (14) and Myers and Montgomery (8) demonstrated lesser debris extrusion when canals were instrumented 1 mm short of the apical foramen. Beeson *et al.* (15) reported that, when the instrumentation was performed upto the apical foramen, significantly more debris was forced apically than when instrumentation was 1 mm short.

*E. faecalis* ATCC 29212 was chosen as the bacteriological marker in this study. It is a non-fastidious, easy-to-grow aerobic bacterium and most commonly found in root canals (16).

Crown-down technique was used in the present study. Initial preparation of the coronal section of the root canal system helps to reduce the number of microorganisms that may be pushed apically (17). Also early flaring of coronal part of the preparation may improve instrument control during preparation of the apical third of the canal (18).

Rotary Protaper extruded less debris and irrigant than K-files. In case of rotary Protaper early flaring of the coronal part of the preparation improved instrument control during preparation of the apical third of the canal. According to Goerig *et al.* (18) rotary motion tends to direct debris towards the orifice, avoiding its compaction in the root canal. In case of K-files, the filing action acts as a piston which may be the reason for more apical extrusion of debris that tends to push the debris through the foramen and less space is available to flush it out coronally (19).

The Protaper systems (hand and rotary) have a progressive taper and a modified guiding tip. Greater cutting efficiency is achieved by the reduced contact area between the dentin and the cutting blades due to its convex triangular cross sectional design. Their design also favors debris removal and prevent the instrument from screwing into the dentinal walls of the canal. One of the significant advantage of the Protaper system is less number of instruments thereby saving time and operator fatigue (20).

The Hand Protaper file prepares the apical area for an extended period of time and the rotational movement of the file is an “operator controlled variable factor” thus leading to more amount of debris, irrigant and bacteria extruding from the canal compared to the rotary protaper which contacts the apical area for a lesser period of time and also the rotational speed and torque is fixed (1,3,21).

Crown-down technique is found to extrude less debris apically compared to the step-back technique (22) and a linear filing motion extrudes more debris when compared to instruments used in rotational motion (8).

It is found that bacteria also extrude along with debris through the apical foramen (17,21-23,24) and thus directly correlates with the weight of the debris (quantitative factor) and the virulence of the bacteria is related to the severity of the periapical inflammation (qualitative factor). As the debris and irrigant extruded by rotary protaper is less in comparison to K-file system, the extruded bacteria are also significantly less. Extrusion of irrigants and debris during canal instrumentation is thus an issue that needs to be controlled (25). The results of this study demonstrated reduced extrusion of bacteria and irrigants by the Protaper rotary compared to other two systems (*p* < 0.00).

Understanding the amount of debris extruded by each instrument system is very essential for the practitioners which can probably be made the basis for selection of a particular instrument system.

## Conclusions

Under the conditions of this *in vitro* study, it may be concluded that the amount of apical extrusion of irrigant solution, bacteria and debris are significantly greater with K-File instruments than Protaper hand and Protaper rotary instruments.

Rotary instruments with high taper used in a crown-down manner produce less extrusion than hand instruments used conventionally. The best way to minimize the extrusion of debris is by adapting crown down technique. Use of engine - driven nickel - titanium instrumentation techniques (Protaper roatary) significantly reduces the apical extrusion of irrigant, debris and bacteria.
